# The Molecular and Histopathological Assessment of Inflammatory Status in Very and Extremely Premature Infants: A Prospective Study

**DOI:** 10.3390/children10020352

**Published:** 2023-02-10

**Authors:** Claudia Ioana Borțea, Ileana Enatescu, Manuela Pantea, Mirabela Dima, Emil Radu Iacob, Catalin Dumitru, Alin Popescu, Florina Stoica, Rodica Elena Heredea, Daniela Iacob

**Affiliations:** 1 Department of Neonatology, “Victor Babes” University of Medicine and Pharmacy Timisoara, Eftimie Murgu Square 2, 300041 Timisoara, Romania; 2 Department of Pediatric Surgery, “Victor Babes” University of Medicine and Pharmacy Timisoara, Eftimie Murgu Square 2, 300041 Timisoara, Romania; 3 Department of Obstetrics and Gynecology, “Victor Babes” University of Medicine and Pharmacy Timisoara, Eftimie Murgu Square 2, 300041 Timisoara, Romania; 4 Department of Ophthalmology, “Victor Babes” University of Medicine and Pharmacy Timisoara, Eftimie Murgu Square 2, 300041 Timisoara, Romania; 5 Department of Pathology, “Louis Turcanu” Children’s Clinical Emergency Hospital, 300041 Timisoara, Romania

**Keywords:** prematurity, preterm birth, neonatal intensive care, inflammatory markers, inflammation

## Abstract

Prematurity comes with a varying range of complications, implying a high prevalence of complications and mortality and depending on the severity of prematurity and the sustained inflammation among these infants, which recently sparked an important scientific interest. The primary objective of this prospective study was to establish the degree of inflammation in very (VPIs) and extremely preterm infants (EPIs) in association with the histology findings of the umbilical cord (UC), while the secondary objective was to study the inflammatory markers in the neonates’ blood as predictors of fetal inflammatory response (FIR). A total of thirty neonates were analyzed, ten of them being born extremely premature (<28 weeks of gestation) and twenty very premature (28–32 weeks of gestation). The EPIs had considerably higher levels of IL-6 at birth than VPIs (638.2 pg/mL vs. 151.1 pg/mL). The CRP levels at delivery did not vary substantially across groups; however, after days, the EPIs had significantly higher CRP levels (11.0 mg/dL vs. 7.2 mg/dL). In contrast, the LDH was considerably higher in the extremely preterm infants at birth and four days after birth. Surprisingly, the proportions of infants with pathologically increased inflammatory markers did not differ between the EPIs and VPIs. The LDH increased considerably in both groups, although the CRP levels increased exclusively among the VPIs. The stage of inflammation in the UC did not vary substantially between the EPIs and VPIs. The majority of infants were identified with Stage 0 UC inflammation (40% in EPI vs. 55% in VPIs). There was a substantial correlation link between gestational age and newborn weight and a significant inverse correlation among gestational age and IL-6 and LDH levels. There was a strong negative association between weight and IL-6 (rho = −0.349) and LDH (rho = −0.261). The stage of the UC inflammation demonstrated a statistically significant direct connection with IL-6 (rho = 0.461) and LDH (rho = 0.293), but none with the CRP. Further studies involving a bigger population size of preterm newborns are required to validate the findings and analyze more inflammatory markers, while prediction models on inflammatory markers that are measured expectantly, before the onset of preterm labor, need to be created.

## 1. Introduction

The death rate for very premature newborns has decreased thanks to advancements in newborn care [1]. Despite this, there is still a significant danger of detrimental effects on neurodevelopment due to various inflammatory reactions occurring in very early deliveries [2,3]. There are extensive neutrophilic infiltrates in acute placental inflammation. These abnormalities, which comprise chorioamnionitis, funisitis, and chorionic vasculitis, are a reaction to inflammation in the amniotic sac [4]. Chorioamnionitis emphasizes the female inflammation, while funisitis emphasize the inflammatory reaction of the fetus. Inflammation of the umbilical veins (vasculitis) and perivascular tissue of the UC comprises the fetal inflammatory reaction to infection (funisitis) [5,6]. In addition to inflammation of the UC arteries, inflammation of the umbilical vein (phlebitis) is also possible when there is no of inflammation of the umbilical arteries (arteritis). A persistent infection might result in tissue necrosis and cellular waste buildup (necrotizing funisitis). Peripheral funisitis, an inflammation of the surface of the UC, may be the earliest indication of an inflammatory reaction [7].

In preterm deliveries, the frequency of histologically diagnosed chorioamnionitis is roughly fifty percent, and its prevalence is negatively correlated to the mother’s gestational age [8,9]. Chorioamnionitis might potentially bring on premature birth since it triggers an inflammatory reaction in the mother. It is also possible for the developing brain to sustain damage from sensitivity to inflammation, and this is especially the case when histologic chorioamnionitis is coupled with fetal inflammatory reaction (FIR) [10,11,12]. Since these veins are connected with the fetal circulation, any sign of mural inflammatory reaction in the UC vasculature or the chorionic plate blood vessels can imply the presence of FIR [13,14,15]. Between fifty and seventy percent of premature placentas that have chorioamnionitis on histological examination also have funisitis. Chorionic plate inflammation affects between 6.15% and about 30% of women. According to some estimates, FIR occurs in anywhere between 25 and 40 percent of all premature newborns [16,17,18]. On the other hand, the intensity of the FIR has not been described in a uniform manner. 

Research has been conducted on a wide variety of cytokines and associated inflammatory indicators in correlation with premature birth [19]. Some have been investigated to see whether or not they are associated with FIR. The inflammatory markers IL-6 and IL-8 are the ones that have been linked to clinical and histologic chorioamnionitis the most often [20]. Additional indicators of infection include interleukin-1 beta and tumor necrosis factor beta. It has not been documented how sensitive these indicators are in comparison to other immunologic markers when it comes to identifying placental inflammatory alterations. Other possible FIR indicators include enzymes and receptors that participate in the cytokine cascade as chemokines and signaling molecules [21,22]. The inflammation in the UC and FIR is both strongly connected with fetal complications, such as early onset neonatal sepsis (EONS) and chorioamnionitis due to maternal inflammatory response [6,23,24]. As a result, it is conceivable that both the magnitude of the FIR or the frequency of EONS will grow in proportion to the development of an UC inflammatory process. Nevertheless, there have only been a few of investigations that have looked at how the amplitude of the FIR relates to the development of inflammation in the UC [25,26]. In addition, their findings were conflicting among research findings, which prevented them from concluding that the FIR continuously increases based on the development of inflammatory process in the UC from umbilical phlebitis via engagement of an umbilical artery that can extend further into Wharton Jelly. It is important to mention that most research had limitations in either the categorization of UC inflammatory processes or the studied population [27,28].

This study aims to analyze the correlation among FIR in the context of preterm birth, as assessed by histochemical analysis of the umbilical cord, associated or not with funisitis. The main goal was to determine the level of inflammation of very and extremely premature neonates in correlation with the histological findings of the umbilical cord, while the secondary objective was to analyze the inflammatory markers from the neonates’ blood, as determinants for FIR. 

## 2. Materials and Methods

### 2.1. Study Design and Ethics

This prospective research project was performed at the Maternity Hospital of the “Pius Brinzeu” County Emergency Clinical Hospital Timisoara and in the neonatal intensive care unit (NICU), at the “Louis Turcanu” Children’s Emergency Clinical Hospital Timisoara, which are associated with the “Victor Babes” University of Medicine and Pharmacy Timisoara (UMFVBT). UMFVBT is governed by the local committee on ethics, which authorizes scientific research based on the International Conference on Harmonization from Helsinki about technical standards for research projects involving humans. The research was conducted in accordance with the ethical norms of the institutions where the study was performed. On 8 January 2021, protocol number 3 was granted clearance to conduct the current investigation. The duration of the study spanned for two years, from January 2021 until January 2023. 

### 2.2. Inclusion Criteria and Study Variables

Premature delivery labor was described as the development of continuous uterine contractions happening at a frequency of at least two per 10 min, accompanied by a modification in the cervical morphology, accompanied by birth before 37 full weeks of gestation. After receiving parental or legal guardian approval, newborns were enrolled in the research. After clearance by the Research Ethics Committee at UMFVBT, the informed consent form was used in accordance with ethical standards and research principles. The study was conducted in a manner that is safe for the environment, and without any harm to the patient, or genetical engineering being involved. Premature newborns delivered vaginally, with a gestational age of less than 32 weeks (31 weeks and 6 days of amenorrhea) admitted to the neonatal intensive care unit will be included in the study. Birth weight was not considered as an exclusion criterion, but the gestational age of 22 weeks was considered as the lower limit for fetal viability. Very preterm newborns were considered from 28 to 32 weeks of gestation, while extremely premature newborns were those born earlier than 28 weeks [29]. 

The primary objective was to demonstrate the existence of fetal inflammation in preterm deliveries; therefore, it was necessary to obtain an umbilical cord segment from the proximal 5 cm from the neonate, which was obtained in the neonatal/maternity ward, independent of maternal disease. Therefore, no data on maternal pathology are presented in the current study. The interleukin 6 (IL-6), C-reactive protein (CRP), and lactate dehydrogenase (LDH) levels were also used to evaluate fetal inflammation, in correlation with the histopathologic evaluation of the umbilical cord. 

The variables taken into consideration included the neonates’ baseline characteristics such as the weight (grams), weight range, gender (male, female), gestational age (weeks), inflammatory markers (interleukin 6, C-reactive protein, lactate dehydrogenase), umbilical cord inflammation stage, and fetal inflammatory response stage.

### 2.3. Study Methods

The following procedures were performed for each neonate included in the study: (1) Umbilical cord histochemical analysis, including umbilical cord segment sampling (approximately 10 cm) from the distal third of the umbilical cord (a maneuver that was performed at birth, in the delivery room or in the operating room); preservation of the segment in aqueous formaldehyde solution (formalin) for transport to the pathology laboratory; fixation of the retrieved sample in paraffin; microscopic examination of umbilical cord sections, performed by the same pathologist. (2) Blood collection for determination of C-reactive protein, IL-6, and LDH at birth, from venous blood, and at 4 days postnatally, also from venous blood. The cord blood was extracted using a needle and placed in EDTA tubes. After collection, blood was held vertically at +4 degrees Celsius in sealed tubes without agitation until analysis.

The histopathology of umbilical cord inflammation correlated with FIR was established as follows, based on existing guidelines [30,31,32]: (1) umbilical phlebitis with neutrophils in the umbilical vein wall was considered as early Stage 1 inflammation; (2) umbilical arteritis, defined as the presence of neutrophils in the wall of one or both umbilical arteries, was considered as moderate level of inflammation, or Stage 2 inflammation; and (3) necrotizing funisitis described as panvasculitis, involving all three umbilical vessels, accompanied by the presence of cellular and neutrophilic debris in the perivascular tissue, sometimes with the appearance of concentric arches of necrosis, was identified as late inflammation, or Stage 3 inflammation. Exposure of the umbilical cord to meconium is often accompanied by mild to severe inflammation manifesting as umbilical phlebitis.

Regarding the IL-6 levels in association with the fetal inflammatory response, it was considered that FIR Stage 1 was determined by the presence of acute funisitis and an umbilical cord blood IL-6 under 11 pg/mL. FIR Stage 2 was identified by a cord blood IL-6 concentration over 11 pg/mL, while FIR Stage 3 corresponds with IL-6 levels higher than 11 pg/mL and the appearance of concentric rings of fetal neutrophils and/or cellular waste surrounding a minimum of one of the umbilical vessels [33,34]. The normal value for IL-6 in the study population was considered for values below 6.6 pg/mL. The CRP was below 5 mg/L, while the normal range for LDH was 120–246 U/L, according to the study laboratory. 

The inflammatory process of the UC was identified using earlier reported guidelines with the existence of neutrophil infiltration into the umbilical vascular walls as follows [32,35]: (0) at least one focus of >5 neutrophils in the UC was considered as Stage 0; (1) Stage 1 inflammation was considered as between 5 and 10 neutrophils in the arterial wall; (2) Stage 2 comprised more than 10 neutrophils surrounding the UC vessels; (3) Stage 3 was considered when more than one focus of at least 5–10 neutrophilic collections was identified and IL-6 levels were below 11 pg/mL; (4) Stage 4 was diagnosed when there was diffuse inflammation with fibrin deposits, or diffuse inflammation, neutrophilic infiltration, and IL-6 levels were above 11 pg/mL.

### 2.4. Statistical Analysis

Using a convenience sample method for an incidence of less than 1% of extreme prematurity in the general population [36], a margin of error of 5%, and a confidence level of 99%, we estimated that a total of 27 subjects are sufficient for a proper statistical power. Nevertheless, due to the lengthy and costly procedure of the histopathological examination and the lack of funding, only 30 samples were analyzed. Data normality was tested with the Kolmogorov–Smirnov test. Normally distributed data were presented by the mean value, as representative for central tendency, and the standard deviation as a measure of dispersion. The difference between the means of the two comparison groups was tested with the Student’s t-test. Proportions were presented as n(%) and compared with the Chi-square test or the Fisher’s exact test, if the frequency assumption was not met for the Chi-square test. The correlation analysis was described using Spearman’s and Pearson’s “rho” correlation coefficients. The test significance was described as “*p*-value”, where a “*p*-value” lower than 0.05 was considered statistically significant. 

## 3. Results

### Patients’ Background Characteristics

Among the 30 participants, ten infants were extremely premature (EPIs) and the other twenty were very preterm infants (VPIs). As expected, it was observed that the average weight was significantly lower among extremely premature newborns (871.5 g vs. 1502.0 g). Regarding the weight range, 70% in the EPI group were born with a weight between 500 and 1000 g, while in the VPI group 55% were born with a weight ranging between 1000 and 1500 g. There were in total 16 male infants and 14 females, as seen in Table 1. Among the extremely premature infants, 20% were born at 24 and 25 weeks of gestation. On the other side, 85% of the very preterm infants were born at 30 and 31 weeks of gestation. 

Table 2 presents the comparison of inflammatory markers by level of prematurity. It was observed that the IL-6 levels measured at birth were significantly more increased among extremely premature infants, compared with the very premature (638.2 vs. 151.1 pg/mL, *p*-value < 0.001). The CRP levels at birth did not differ significantly between groups, but after 4 days the CRP increased significantly higher among the very premature infants (11.0 mg/dL in the VPI group vs. 7.2 mg/dL in the EPI group, *p*-value < 0.001). On the other hand, the lactate dehydrogenase was significantly more elevated among the extremely premature, both at birth and 4 days after birth. Surprisingly, the proportions of infants with pathologically increased inflammatory markers did not differ between the EPIs and VPIs, regardless of IL-6, CRP, or LDH.

The comparison of inflammatory markers measured at birth and 4 days after birth between extremely premature newborns and very premature newborns is presented in Table 3. The mean LDH levels increased significantly after four days in the EPI group (851.8 UI/L vs. 962.3 UI/L, *p*-value = 0.003) and in the VPI group (468.9 UI/L vs. 565.9 UI/L, *p*-value = 0.010), respectively. However, the CRP levels increased significantly only among the very preterm infants (4.6 mg/dL vs. 11.0 mg/dL, *p*-value < 0.001), although those in the EPI group were born extremely preterm.

**Figure 1:**
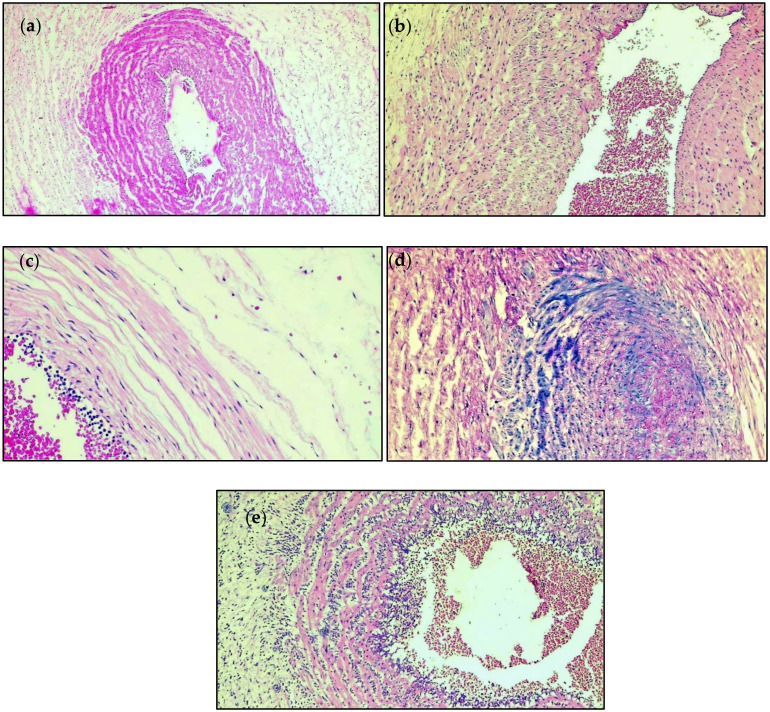
Histopathological findings; (**a**)—Stage 0 UC inflammation; (**b**)—Stage 1 UC inflammation; (**c**)—Stage 2 UC inflammation; (**d**)—Stage 3 UC inflammation; (**e**)—Stage 4 UC inflammation.

Table 4 describes the comparison of histopathology findings from the umbilical cord of newborns by degree of prematurity. It was observed that the stage of inflammation did not differ significantly between the extremely premature infants (born under 28 weeks of gestation) and very premature infants that were born between 28 and 32 weeks of gestation. The majority of newborns were diagnosed with Stage 0 inflammation at the site of the umbilical cord (40% in the EPI group vs. 55% in the VPI group). There were 4 (40%) infants in the EPI group with S3 and S4 inflammation, compared with 6 (30%) in the VPI group. Several histopathological findings from the study groups’ samples are described in Figure 1. Similarly, 50% of the EPI infants were identified with Stage 1 FIR, compared to 65% in the VPI infants, without any significant differences. 

**Figure 2:**
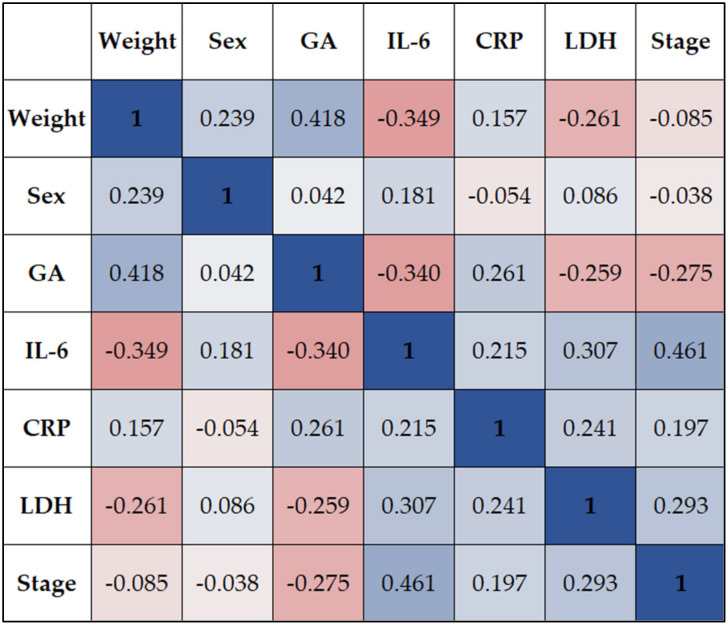
Correlation matrix; GA—Gestational Age; IL-6—Interleukin 6; CRP—C-reactive Protein; LDH—Lactate Dehydrogenase; Stage—Umbilical cord degree of inflammation.

Lastly, the correlation analysis presented in Table 5 and Figure 2 identified multiple statistically significant associations between the study variables. Gestational age had a significant positive association with the infants’ weight (rho = 0.418), and a significant negative correlation with the IL-6 (rho = −0.340) and LDH (rho = −0.259) levels. Similarly, the infants’ weight had a significant negative correlation with IL-6 (rho = −0.349) and LDH (rho = −0.261). The stage of UC inflammation had a significantly direct correlation with IL-6 (rho = 0.461) and LDH (rho = 0.293), although no association with the CRP. 

## 4. Discussion

### 4.1. Literature Findings

The current study determined an important association between the level of prematurity defined by gestational age and the degree of inflammation of the neonate described by the FIR. However, the association of an UC inflammatory process, as described by the inflammatory cells from the UC samples and the inflammatory cells identified in the UC at the moment of birth (funisitis), was not statistically significant. In the studied patients with funisitis, our results revealed alterations in oxidative and inflammatory mediators in cord blood as a result of the prenatal FIR, associated with a very preterm birth or extreme prematurity. 

The current study proposed to histologically analyze the umbilical cord instead of measuring only the inflammatory markers of the newborn, due to the difference in mechanisms that trigger FIR, previously documented by Para et al. [33]. The researchers discovered that FIR type I was defined by an increase in host immunological responses, including neutrophil and monocyte activities, a cytokine storm, and a decrease in T cellular functions. Type II FIR was characterized by a modest chronic inflammatory response reflecting fetal transplant rejection. RNA research indicated that monocytes, macrophages, and neutrophils were primarily responsible for FIR type I immunological responses.

Similarly, another recent study attempted to quantify the level of inflammation among preterm newborn patients in comparison with full-term newborns [37]. Preterm newborns had considerably greater quantities of oxidative stress and funisitis relative to full neonates in the comparison group. These findings may suggest that the FIR in funisitis is linked with a more pronounced inflammatory and oxidative response in the infant than in individuals with chorioamnionitis. In contrast, our research examined only the presence of funisitis and not chorioamnionitis. Newborns are more vulnerable to oxidative tissue damage and have a higher risk for oxidative stress [38]. This is due to the fact that infants have poorer physiological defenses against oxidation and, relative to healthy adults, newborns also have reduced amounts of antioxidants. The causes of the disparity between septic and inflammatory diseases in people remain unknown after much investigation. Under normal physiological settings, there is a homeostatic equilibrium between the synthesis and clearance of endogenous antioxidant molecules, which is interrupted by inflammation [39].

Another study attempted to observe the molecular basis of the inflammation process that happens within the umbilical cord in the premature neonates, but the research was performed in vitro [40]. The researchers examined the cytokine pattern of UC blood leukocytes activated with lipopolysaccharides of vaginal bacteria often identified in pregnant women who had a history of preterm birth. The experiment was carried in healthy tissue from infants of donor mothers in good health. Intriguingly, the capacity of the UC to react to the inflammatory activity differed significantly across donors, indicating a substantial individual variability in the induced immune response, with IL-6 showing the greatest activation, with levels reaching over 200,000 pg/mL.

The current research implies that monitoring cytokine concentrations might aid in the identification of infections and other inflammatory processes that might be involved in the onset of a premature labor. Other studies have shown that it is feasible to quantify inflammation markers in amniotic fluid obtained non-invasively from the vaginal fluids of women experiencing preterm labor. High levels of IL-6 in amniotic fluid accurately predicted the FIR [41]. By incorporating the umbilical cord as a noninvasive source of newborn plasma, as in other investigations, it may be possible to determine the cytokine profile of infants and assess their risk of infection-related problems after delivery.

Combining anti-inflammatory medication and antimicrobials has been recommended to address intra-uterine infections and minimize inflammatory responses that contribute to preterm labor and unfavorable fetal outcomes. The particular combination of corticosteroids and NSAIDs exemplifies how appropriate therapies improve the survival of preterm newborns by increasing lung maturation, but also decrease the level of inflammation that can reduce the risk of FIR. Similar to betamethasone monotherapy, another research demonstrated that the combination treatment successfully lowered the production of the pro-inflammatory cytokines [42]. Other investigations also revealed that postnatal administration of indomethacin to preterm neonates born before 28 weeks of gestation had positive effects on the fetal brain [43]. This neuroprotective action of anti-inflammatory medication might be caused by a rise in stability of cerebral hemodynamics.

Another recent study comparable to ours was performed recently, with a population size bigger than 200 participants [28]. The researchers attempted to determine if the progression of the UC inflammatory processes is associated with the progression of FIR, and according to regression modeling, the more extensive the evolution of inflammation in the UC (funisitis), the stronger predictive value of presumed newborn sepsis, with Stage 3 level of inflammation having an odds ratio of 11. This research provided evidence that funisitis is both the qualitative and quantitative histopathological equivalent of FIR and a predictive sign for chorioamnionitis. 

Regarding the blood sampling and histopathological analysis of UC samples in our study, it should be mentioned that only one proximal portion of the UC was sampled, without a comparison from a more distal portion. Moreover, the venous sampling for inflammatory markers can influence the results, since the slow blood flow can determine a higher degree of margination of the inflammatory cells, and therefore a higher quantity of inflammatory markers, which was demonstrated in two studies [28,44]. Blanco-Elices et al. demonstrated the presence of histological variations across distinct zones with major histological changes in their matrix and cell composition, confirming the unique capability of each zone as a pool of cells for use in vascular tissue analysis. It has been hypothesized that the UC microenvironment provided by the fibrillar and non-fibrillar components of the matric enables cells to interact and regulates cellular functions and actions; therefore, the unique characteristics of each zone are associated to their unique physical characteristics.

### 4.2. Study Limitations and Future Perspectives

Among the limitations of the current study, there is the lack of a control group to compare the level of inflammatory markers in the umbilical cord and blood. Another limiting factor is the relatively small sample size that reduces the statistical power of our findings. Additionally, clinical observations and newborns’ short-term or long-term outcomes were not considered. As for the future perspectives based on this pilot study, a bigger project is expected to involve preterm newborns between the 32nd and the 37th week of gestation, in comparison with the EPI and VPI infants that were analyzed now. A wider range of inflammatory markers and complications such as prematurity retinopathy will be studied, as well as histopathological analysis of the placenta, besides the umbilical cord. Further studies involving a bigger population size of preterm newborns are required to validate findings and analyze more inflammatory markers. Prediction models can also be created in prospective studies based on inflammatory markers that are measured expectantly, before the onset of preterm labor. 

## 5. Conclusions

The quantity of inflammatory markers and inflammatory cells is greater in the cord blood of neonates as the gestational age decreases. As an early sign of dangerous inflammatory conditions, assessing the inflammatory markers may be useful from the cord blood of the infant, instead of analyzing the UC samples under the microscope, which comes at greater prices and a much longer time needed to analyze the samples. The marker analysis can be conducted in a matter of minutes and provides a good overview of the stage of funisitis and potential complications while helping to decide the further management of the preterm newborn, such as administering corticosteroids and NSAIDs. 

## Figures and Tables

**Table 1:** The characteristics of EPI and VPI infants included in the study.

Variables	Total (*n* = 30)	EPI (*n* = 10)	VPI (*n* = 20)	*p*-Value
Weight, grams (mean ± SD)	1291.8 ± 405.8	871.5 ± 241.6	1502.0 ± 292.2	<0.001
Weight range				<0.001
500–1000 g	8 (26.7%)	7 (70.0%)	1 (5.0%)	
1000–1500 g	14 (46.6%)	3 (30.0%)	11 (55.0%)	
1500–2000 g	8 (26.7%)	0 (0.0%)	8 (40.0%)	
Gender				0.795
Male	16 (53.3%)	5 (50.0%)	11 (55.0%)	
Female	14 (46.7%)	5 (50.0%)	9 (45.0%)	
Gestational age (weeks)				–
24–25	2 (6.7%)	2 (20.0%)	0 (0.0%)	
26–27	8 (26.7%)	8 (80.0%)	0 (0.0%)	
28–29	3 (10.0%)	0 (0.0%)	3 (15.0%)	
30–31	17 (56.6%)	0 (0.0%)	17 (85.0%)	

Data described as *n* (%) and compared using Chi-square test and Fisher’s exact unless specified differently; EPIs—Extremely Premature Infants (<28 weeks of gestation); VPIs—Very Premature Infants (28–32 weeks of gestation); SD—Standard Deviation; mean ± SD compared with Student’s *t*-test (unpaired).

**Table 2:** Comparison of inflammatory markers by level of prematurity.

Inflammatory Markers	Total (*n* = 30)	EPI (*n* = 10)	VPI (*n* = 20)	*p*-Value
IL-6, (mean ± SD)	319.1 ± 78.6	638.2 ± 122.7	151.1 ± 26.7	<0.001
CRP at birth, (mean ± SD)	5.5 ± 1.1	6.0 ± 1.8	4.6 ± 2.6	0.138
CRP at 4 days, (mean ± SD)	10.0 ± 0.9	7.2 ± 3.2	11.0 ± 1.3	<0.001
LDH at birth, (mean ± SD)	596.5 ± 46.3	851.8 ± 72.2	468.9 ± 108.2	<0.001
LDH at 4 days, (mean ± SD)	698.0 ± 45.6	962.3 ± 69.9	565.9 ± 119.0	<0.001
IL-6 (N: <6.6 pg/mL)				0.128
Pathological	26 (86.7%)	10 (100%)	16 (80.0%)	
Normal	4 (13.3%)	0 (0.0%)	4 (20.0%)	
CRP (N: <5 mg/dL)				0.704
Pathological	26 (86.7%)	9 (90.0%)	17 (85.0%)	
Normal	4 (13.3%)	1 (10.0%)	3 (15.0%)	
LDH (N: 120–246 UI/L)				0.472
Pathological	29 (96.7%)	10 (100%)	19 (95.0%)	
Normal	1 (3.3%)	0 (0.0%)	1 (5.0%)	

Data described as *n* (%) and calculated using Chi-square test and Fisher’s exact unless specified differently; EPI—Extreme prematurity (<28 weeks of gestation); VPI—Very Preterm (28–32 weeks of gestation); SD—Standard Deviation; IL-6—Interleukin 6; CRP—C-reactive Protein; LDH—Lactate Dehydrogenase; mean ± SD compared with Student’s *t*-test (unpaired).

**Table 3:** Comparison of inflammatory markers measured at birth and 4 days after birth between extremely premature newborns and very premature newborns.

	EPI (*n* = 10)			VPI (*n* = 20)		
Markers	At Birth	At 4 Days	*p*-Value	At Birth	At 4 Days	*p*-Value
CRP (mean ± SD), mg/dL	6.0 ± 1.8	7.2 ± 3.2	0.315	4.6 ± 2.6	11.0 ± 1.3	<0.001
LDH (mean ± SD), UI/L	851.8 ± 72.2	962.3 ± 69.9	0.003	468.9 ± 108.2	565.9 ± 119.0	0.010

EPI—Extreme prematurity (<28 weeks of gestation); VPI—Very Preterm (28–32 weeks of gestation); SD—Standard Deviation; CRP—C-reactive Protein; LDH—Lactate Dehydrogenase; mean ± SD compared with Student’s *t*-test (paired).

**Table 4:** Comparison of histopathology findings by level of prematurity.

Level of Inflammation	EPI (*n* = 10)	VPI (*n* = 20)	*p*-Value
UC stage of inflammation			0.902
S0	4 (40.0%)	11 (55.0%)	
S1	1 (10.0%)	1 (5.0%)	
S2	1 (10.0%)	2 (10.0%)	
S3	2 (20.0%)	4 (20.0%)	
S4	2 (20.0%)	2 (10.0%)	
FIR			0.606
Stage 1	5 (50.0%)	13 (65.0%)	
Stage 2	2 (20.0%)	4 (20.0%)	
Stage 3	3 (30.0%)	3 (15.0%)	

Data described as *n* (%) and calculated using Chi-square test and Fisher’s exact unless specified differently; UC—Umbilical Cord; FIR—Fetal Inflammatory Response; EPI—Extreme prematurity (<28 weeks of gestation); VPI—Very Preterm (28–32 weeks of gestation).

**Table 5:** Correlation analysis of the studied variables.

		Weight	Sex	GA	IL-6	CRP	LDH	Stage
Weight	Rho	1	0.239	0.418 **	−0.349 **	0.157	−0.261 *	−0.085
	*p*-value	-	0.106	0.003	0.003	0.230	0.022	0.087
Sex	Rho	0.239	1	0.042	0.181	−0.054	0.086	−0.038
	*p*-value	0.106	-	0.378	0.244	0.423	0.624	0.466
GA	Rho	0.418 **	0.042	1	−0.340 *	0.261	−0.259 *	−0.275
	*p*-value	0.003	0.378	-	0.038	0.056	0.030	0.063
IL-6	Rho	−0.349 **	0.181	−0.340 *	1	0.215 **	0.307 **	0.461 **
	*p*-value	0.003	0.244	0.038	-	0.000	0.002	0.000
CRP	Rho	0.157	−0.054	0.261	0.215 **	1	0.241 *	0.197
	*p*-value	0.230	0.423	0.056	0.000	-	0.027	0.064
LDH	Rho	−0.261 *	0.086	−0.259 *	0.307 **	0.241 *	1	0.293 **
	*p*-value	0.022	0.624	0.030	0.002	0.027	-	0.006
Stage	Rho	−0.085	−0.038	−0.275	0.461 **	0.197	0.293 **	1
	*p*-value	0.087	0.466	0.063	0.000	0.064	0.006	-

** Correlation is significant at the 0.01 level (2-tailed); * Correlation is significant at the 0.05 level (2-tailed); GA—Gestational Age; IL-6—Interleukin 6; CRP—C-reactive Protein; LDH—Lactate Dehydrogenase.

## Data Availability

Data available on request.
